# Supplementary material for: Are carbon emissions trading and green financial instruments synergistic? -Comprehensive quantitative research based on content analysis
Source: PLoS One. 2024 Mar 7;19(3):e0298601. doi: 10.1371/journal.pone.0298601 (PMC10919601; doi:10.1371/journal.pone.0298601)
Supplement: S1 Data — (ZIP) [file pone.0298601.s001.zip › Data/地级市Z_F工作报告.zip!/║ú─╧/║ú┐┌╩╨/└┤╫╘╣1⁄2╓┌║┼í╛┬φ┐╦╩2╛▌═°í┐_2004.docx]

各位代表：

现在，我代表市人民政府，向大会作政府工作报告，请予审议，并请各位政协委员和其他列席人员提出意见。

一、2003年政府工作回顾

万众耕耘初局开，春华秋实硕果来。2003年，新一届政府在中共海口市委的正确领导下，以“三个代表”重要思想总揽全局，以“两个翻番,两个率先”为总目标，奋力拼搏，励精图治，开拓创新，扎实工作，经过全市人民的共同努力，全面完成了市十三届人民代表大会第一次会议确定的各项工作目标，实现了新一届政府工作的良好开局。

经济协调健康快速发展，整体质量效益明显提高。2003年，实现全市生产总值238.18亿元，同比增长13.6%，创下1993年以来经济增长速度的新高。其中，第一、二、三产业分别实现增加值26.1亿元、79.75亿元和132.33亿元；分别增长6.1%、25.9%和8.7%。财政总收入42.9亿元，增长31.3%，增长幅度位居全省第一。年末金融机构各项贷款余额514.09亿元，比年初增长19.4%，年末金融机构各项存款余额618.55亿元，比年初增长21.6%，金融业对经济发展的支持力度增强。固定资产投资104.99亿元，增长12.1%。实现工业总产值220.57亿元，增长39.5%，工业对经济增长的贡献率达到47.9%，工业“两税”对财政收入增长的贡献率达到58.3%，成为推动我市经济快速发展的主导力量。经过几年来的经济结构调整，全市经济正在向三次产业相协调，规模结构相优化，速度质量相统一，经济社会可持续发展的态势转变。优势主导产业日益明显，经济增长的产业基础进一步增强，我市已进入了以工业为主导的新一轮经济社会全面快速协调发展时期。

经济总量不断扩大，优势产业稳步发展。以扩大经济总量为目标，创新工作思路，全面推进重点企业、重点项目建设，形成了工业总量不断扩大，旅游活力不断增强，商业贸易不断搞活的良好局面。实施“五强”计划，扶持中小企业发展，有力地促进了工业经济总量的不断扩大。汽车产值75亿元，增长159%；交通运输设备制造业产值78.7亿元，增长1.2倍；医药制造业产值21.5亿元，增长24.4%；食品饮料业产值41.8亿元，增长11%；规模以上工业实现增加值47.34亿元，增长36.8%。创新“五区合一”管理体制，保税区全年实现工业总产值22亿元，增长37.5%。工业重点项目建设有新进展，共推出71个重点项目，合同总投资达85.53亿元，21个项目建成投产；“药谷”计划实施进展顺利，发展后劲不断增强。在“阳光岛、健康城、生态市”品牌效应的作用下，旅游业在非典过后呈现恢复性增长。接待过夜国内外游客394万人次，增长0.7%。商业建设投资增大，步伐加快，新增商业网点面积12万平方米，一批商贸业重点项目正在兴建。会展经济催旺市场，全年完成社会消费品零售总额86.14亿元，增长11.3%；港口货物吞吐量达到2100万吨，增长17.2%；邮电业务总量达到21.14亿元，增长20.3%。

高标准规划新海口取得重大进展，高起点建设新海口取得初步成效。创新规划理念，在全国省会城市首次进行了城市总体规划概念国际招标、总体规划纲要国内招标、“海口外滩”城市设计国际招标、西海岸规划设计国际招标，在省内外、国内外引起了广泛的高度关注，提高了城市的知名度。在此基础上，启动了城市生态环境建设规划、园林绿化规划、水系规划、路网规划、城市灯光规划等五个专业性规划。加快推进城市基础设施重点项目建设，在基本完成以“一河两桥三路”为代表的十大工程建设的基础上，启动“3366”工程项目建设，推进秀英大道街区、金贸街区等城区改造。停缓建工程处置取得重大进展，商品房销售96.17万平方米，增长69.4%。开展“八查十治”专项整治活动，城市卫生环境明显改善。启动城市周边生态森林工程建设，推进“五大出口”植树造林工程，全年“三边林”造林1万多亩。城市建设步入强化功能、提升品位、突出特色并举的新阶段，基本实现了“一年一小变”的目标。

“三农”工作得到高度重视，农村经济快速发展。按照“城乡互动，三次产业联动”的指导思想，着力调整农业产业结构。以加工业为龙头，加快水产品出口加工基地和100万头生猪生产加工项目建设。推进种植结构的调整优化，推广无公害蔬菜生产，全年种植冬季瓜菜约13万亩。加强植树造林工作，全年植树造林突破5万亩。加强农田水利综合整治，在全省冬修检查评比中荣获第一名。积极组织冬交会工作，在冬交会评比中获第一名。农业在全省的各项评比中共获得六项大奖，一举改变了海口农业在全省的形象。积极稳妥地推进农村税费改革，农民人均负担减少88.3%。2003年，“三农”工作基本实现了工作思路、工作目标、结构调整的三大转变，我市农业正向着城郊型、服务型、加工型、高科技型方向转变。农村经济协调发展，全年完成农业总产值36.72亿元，增长6.5%；农民人均纯收入3271元，增长6.0%，实现了农业增效，农村发展，农民增收。

实施最强措施防治非典，抗击非典取得双胜利。在抗击非典的战斗中，我们坚决贯彻党中央、国务院和省委、省政府的决策和部署，以对人民群众身体健康和生命安全高度负责的精神，以临战状态，战时标准，全市动员，全民动手，建立健全城乡一体的非典防治工作组织领导体系，构建全覆盖的群防群控网络，采取了关口前移，重点布控，双零报告，及时隔离等措施防治非典。全市人民团结一心，众志成城，严防死堵，用最强的措施防治非典，下最大的力气抓经济建设，保住了海口无疫区，取得了抗击非典和经济建设的双胜利，为树立“海南健康岛”品牌作出了重大贡献，为实现海口可持续发展创造了新的亮点。

体制创新步伐加快，招商引资成效明显。推进行政管理体制改革，完成了城市环卫管理决策权、执行权、监督权三权分离试点改革。深化行政审批制度改革，取消了一系列不是法定的程序和职能。在深化、完善“三制”的基础上，启动了行政审批中心的筹建工作。完善城市经营体制改革，实行城市公共资源的市场化运作，出租汽车、机动车牌号、公交线路、广告经营权公开拍卖的总收益突破1亿元。深化城市基础设施投融资体制改革，成功与国家开发银行合作，初步建立了“政府入口、市场出口”的城市建设投融资体制的基本框架。深化国有企业改革，完成了市电子公司、橡胶五厂等的改制任务，3200多名员工得到了妥善安置。基本完成了市税务部门、琼山工商部门的改革。推进了开发区企业、农口企业的改革。认真落实土地交易体制改革，全面实行了经营性土地的招标拍卖挂牌出让。加强国有土地整治，加大对闲置土地的依法回收力度，开展农村土地确权工作。推进市政工程监督改革，组建市政工程监督小组，强化市政工程的审计监督。创新招商引资方式，抓住“今日无非典，明日是亮点”的机遇，成功举办“海口药谷”招商会，签约项目14个，合同总投资78亿元。坚持走出去招商，先后在北京、深圳、东莞等地成功举办重大招商活动，签约项目63个，合同总投资额148.83亿元人民币和1.86亿美元。其中，已动工、落户的项目有52个，正在推进的项目有6个。日本住友、韩国三星、三九集团、万全科技、先声集团、鲁能集团等大企业纷纷落户海口。

社会各项事业协调发展，精神文明建设水平不断提高。科技发展不断进步，海口生物制药基地被国家科技部认定为区域类“十五”国家“863”计划成果产业化基地。教育体制改革不断深化，优质教育不断扩大，基础教育不断加强，各类教育全面发展，完成2.5万平方米的中小学D级危房改造。加快公共卫生体系、防疫基础设施和重点医院建设，城乡卫生条件得到改善，社区卫生服务被列为国家试验区。双拥工作取得新成绩，第五次荣获“全国双拥模范城”荣誉称号。文学艺术创作硕果累累，一批优秀作品获国家、省级奖励。体育事业喜获丰收，参加全国城运会创历届最好成绩，群众性文体活动蓬勃发展。人口与计划生育工作得到加强，全面完成了计划生育的各项任务。开展“告别陋习，珍爱健康，保护家园”一系列活动，塑造和展示了海口的文明新形象。文明生态村创建活动成效显著。外事侨务、对台事务、人民武装、民族宗教、老年人、妇女儿童等工作都取得了新的成绩。加强社会治安综合治理，妥善处理各类群体性事件，全市政治安定，社会稳定，政通人和。

执政为民扎扎实实办好实事，城乡联动人民生活稳步提高。坚持访贫问苦制度，健全援助网络，认真做好特困居民的援助工作，解决特困居民的“六难”问题成效显著。15892名特困居民纳入低保救济，5400名特困子女获得就业就学援助，269名特困户住上了解困房，3200名特困居民用上了就医优惠卡。千名干部下乡扶贫工作有新成效，千里乡村道路建设进展顺利，解决农村22万人饮水困难工程取得重大突破，为民办实事工程基本完成。社会保障机制不断完善，“两个确保”全面落实，三条保障线紧密衔接，3.5万企事业退休人员实现了社区社会化管理。就业再就业工作不断加强,就业服务体系不断完善，就业岗位不断增加，优先解决“4050”人员和“四无”就业困难人员的就业问题，全年实现就业再就业27500人。我们一手抓为民办实事，一手抓统筹区域经济发展。四区经济社会协调发展，城乡面貌出现新变化，人民生活水平稳步提高。在岗职工平均工资增长7.5%，城镇居民人均可支配收入增长4.3%。

政府自身建设不断加强，机关作风明显改善。新一届政府班子以“为民、务实、清廉”为总要求，不断加强自身建设，在实践中熟悉情况，在工作中经受锻炼，下最大的力气抓好工作。认真落实“三个善待”理念，建立了重点项目跟踪服务，现场办公解决实际问题，领导定点联系重点企业，行政优质服务，深入基层调查研究等制度，推行了监督专员评议部门、行政效能监督等工作，开展行政优质服务月活动，坚持从快、从优为企业解决了一系列历史遗留问题和发展中遇到的困难。各级政府领导班子坚持“求真务实”精神，看大局，抓重点，谋发展，紧紧围绕“两个翻番，两个率先”落实工作任务，紧紧围绕发展重点落实建设项目，紧紧围绕重点任务落实工作措施，紧紧围绕为民办实事出实招，使实劲，求实效。促进了机关作风转变，改善了投资环境，树立了政府的良好形象，推进了全市经济社会的全面协调快速发展。

各位代表，过去的一年，是不平凡的一年，成绩来之不易。这一年，是新一届政府在创新中转换角色，在创新中适应工作，在创新中加快发展的一年；是坚持一手抓经济建设这个中心不动摇，一手抓防治非典这件大事不放松，取得“抗非典，促发展”双胜利的一年；是社会稳定，人民安居乐业，城市建设、社会事业取得重大进展的一年。这是省委、省政府和市委正确领导的结果，是全市人民开拓创新、团结拼搏的结果，是全市社会各界和衷共济、共同努力的结果。在此，我代表市政府向全市各族人民、驻市部队、武警官兵、港澳台同胞、海外侨胞、国际友人，向所有关心支持海口建设的投资者和朋友们，表示衷心的感谢并致以崇高的敬意！

为政之忧，忧在困难。我们清醒地看到，当前，海口仍存在一些亟待解决的矛盾和问题：一是影响发展的体制性障碍依然存在，体制创新有待深化，政府职能有待转变，发展环境有待优化；二是经济结构性矛盾仍然突出，经济增长速度与结构、质量与效益不够协调，经济增长基础不够牢固，工业化水平不高，综合实力和竞争力还不够强；三是财政还比较困难，税基不宽，含税产业不丰，财政收入作为政权运转和社会保障仍然存在较大差距；四是城乡居民收入增长缓慢，城乡差距明显，农村脏乱差问题还没有从根本上得到改善，农村城市化进程亟需加快，农业产业化，农村基础设施建设，农村小康建设任重道远；五是城市基础设施建设欠账太多，基本功能仍需配套，社会事业基础设施滞后，公共管理薄弱;六是城市管理水平有待加强, 交通状况有待改善，多头执法、以罚代法的现象仍然存在;七是市民文明素质，城市文明程度亟待提高；八是社会治安仍然存在不稳定因素，群体性上防事件时有发生，街头“两抢”、农村吸毒现象仍然存在；九是“三个善待”理念仍需进一步巩固，公务员整体素质有待提高，公仆服务意识有待加强，办事效率和服务质量有待提高等等。这些矛盾和问题，我们将高度重视，立足长远，着眼当前，统筹考虑，上下努力，在今后工作中，通过改革和发展逐步加以解决。

二、2004年的发展目标、主要任务和总体要求

2004年是实现“两个翻番，两个率先”，全面建设小康社会的关键一年；是贯彻市党代会提出的“做大做强做精做美”新海口，实现“一年一小变，三年一中变，五年一大变”的重要一年。做好今年的工作，意义重大。

今年政府工作的指导思想和总体要求是：以邓小平理论和“三个代表”重要思想为指导，深入贯彻党的十六大、十六届三中全会、省委四届三、四次全会和市第十次党代会精神，树立和落实以人为本，全面、协调、可持续的科学发展观，解放思想，求真务实，以统筹城乡发展，调整经济结构，优化产业环境，创新管理体制，促进全面发展为主题，以深化各项改革、扩大内外开放为动力，以扩大经济总量为重点，全面加快新型工业发展；全面加大城乡基础设施建设；全面加快农村经济发展；全面加快科技、文化、教育、卫生等社会各项事业的发展。不断提高工业化水平；不断提高农业生产力水平；不断提高城市化水平；不断提高城乡居民的生活质量和水平，促进全市经济社会全面、协调、快速、健康发展。

2004年经济社会发展的预期目标是：海口市生产总值增长13%，财政收入增长12%，固定资产投资增长15%，社会消费品零售总额增长11%，城镇居民人均可支配收入增长7.5%，农民人均纯收入增长6.5%，城镇登记失业率控制在3.5%以内，人口自然增长率控制在8‰以内。

实现上述目标，我们必须看到，世界正处在政治多极化、经济全球化趋势加快，全球经济复苏，国际产业转移加快时期。全国经济正处在快速发展阶段。全省经济已进入快速发展期，我市正处在快速发展的重要战略机遇期。同时也应看到，当前消费需求不足，国家采取适度调控的宏观政策和我市经济运行中存在的深层次问题等都必将对我市产生一定的影响。因此，我们必须坚持“五个统筹”、“五个坚持”的方针；必须坚持以人为本，树立全面、协调、可持续的科学发展观；必须坚持规模、速度、结构、质量、效益相统一的原则；必须坚持城乡统筹，区域和经济社会全面协调发展；必须坚持实现好、维护好、发展好人民群众的根本利益；必须坚持社会主义市场经济的改革方向，紧紧抓住当前重要机遇期不动摇，聚精会神搞建设，一心一意谋发展，努力实现全市生产总值的重大突破，努力实现工业总产值的重大突破，努力实现财政收入的重大突破，努力实现城乡基础设施建设的重大突破，为推进我市“三个文明建设”奠定坚实的物质基础，促进全市经济社会与资源环境，人与自然和谐发展。

三、以壮大经济总量为重点，大力发展优势产业，加快工业化发展进程，大力发展民营经济，全面提高海口经济实力和综合竞争力。

扩大海口经济总量的核心是壮大工业经济总量。要充分发挥产业优势、体制优势和环境优势，坚持不污染环境，不破坏资源，不搞低水平重复建设，走科技含量高，经济效益好，资源消耗低，环境污染少，人力资源优势得到充分发挥的新型工业化道路，实现新兴工业的跨越式发展带动相关产业发展，不断壮大全市的经济总量。

加快工业化进程，壮大工业经济总量。推进支柱产业发展，培育完善四大支柱产业为基础的新兴工业体系。做大做强以海汽、新大洲为龙头的机械制造业，加快海汽三、四期工程建设，加快海口国家科技工业园区建设；加快实施“药谷”计划，把海口建成环北部湾及华南地区的药品生产加工基地；加强保税区建设，积极推动韩国三星光缆项目落户海口。做大做强以椰树、椰岛、海南红塔和亚太酿酒为龙头的食品饮料和烟酒制造业，把海口建成我国最大的热带天然果汁加工和出口基地。做大以兴业聚酯为骨干的化纤纺织业，力争16万吨瓶级聚酯项目年内建成投产，把海口建成我国华南地区重要的化纤纺织生产基地。推进重点项目建设，培育和发挥大企业、大集团的骨干带头作用。重点扶持“强优”企业发展，扶持培育一批一流企业，重点发展一批年产值超10亿元，超100亿元的大型工业企业，以大企业、大集团为骨干，形成工业发展的支撑力。积极扶持中小企业发展，特别是要扶持有助于支柱产业和大企业发展的配套企业，夯实工业基础。调整优化生产力布局，实行“五区合一”管理体制，筹划建设汽车工业园，筹建美安工业基地，加快形成以南海大道为轴线，以海口保税区、高科技园区等基地为轴带的工业走廊，发挥海口西部工业走廊的产业优势和辐射作用。

加快特色旅游景点建设，着力提升海口旅游。注重规划，抓好精品景点、景区建设，构建海口精品旅游景区主体框架，加快旅游业发展。抓好红树林生态风景区的规划和火山口国家地质公园的建设。加快西海岸黄金旅游带的建设,努力把西海岸建成休闲旅游度假中心、海上运动中心、高尚娱乐中心。启动“海口外滩”项目建设，把“海口外滩”打造成集休闲、旅游、观光为一体的一条靓丽的风景线。精心设计旅游精品线路，开辟海上观光路线，开设生态旅游专线，整合旅游资源，强化旅游宣传促销, 扩大客源市场。整顿和规范旅游市场，强化行业自律，按照国际标准全面提高行业管理水平和服务质量。

调整优化商业布局，发展现代商贸物流。抓好全市商业网点和各类专业市场的规划建设。以海秀大道为主轴打造海口商务中心区，加快南海大道汽车交易一条街、秀英大道旅游商品服务一条街的建设。在全市推进以海南GSP医药城、龙华区大型农产品物流中心等为代表的一批大型物流商场和重点专业市场的规划建设。清理整治专业批发市场，逐步调整商业网点布局，完善商业网点、专业批发市场等现代物流网络。依托机场、铁路和港口的优势，建设一批综合型货仓，逐步形成服务全省，辐射环北部湾地区的集批发、加工、分装、仓储、转运五大功能为一体的港口现代物流体系。

认真贯彻《决定》精神，大力发展民营经济。全面贯彻《海口市关于加快民营经济发展的决定》，为加快民营企业发展营造宽松的发展环境。进一步放宽民营资本的投资领域和准入范围，允许民营资本进入法律法规未禁入的行业和领域，支持民营资本参与城市建设和旧城区改造。扶持一批核心竞争力强的优势民营企业做大做强，在开拓市场、信息服务、创业导向、人才培训、技术交流、对外合作等方面提供优质服务。建立健全民营企业信用担保体系、诚信评价体系、人才培训体系、法律信息服务体系，着力解决民营企业融资难的问题。为民营经济发展提供更便捷、更优质的服务。

四、以提高农民收入水平为目标，统筹城乡协调发展，加强农业基础设施建设，努力实现农业增产农民增收

离开了农村的小康，就没有全市的小康。要认真贯彻落实中央一号文件，按照“多予、少取、放活”的要求，在财政支出、资源配置、基础设施建设、社会保障、劳动力流动等方面向农村倾斜，从投入机制上解决“三农”问题，促进农村经济快速发展，逐步改变城乡二元结构。

加大农业基本建设投入，为农民增收打好基础。要调整财政支出结构，市区两级财政每年安排2000万元用于农田水利综合整治，每个区每年计划改造两个大田洋，确保五年内投资1亿元，全面改善全市农业的生产条件，提高全市农业生产力水平。切实加强农村教育、卫生、文化基本建设，完成全市农村中小学D级危房改造。继续推进农村人畜饮水工程，加快千里乡村道路建设，改善农村的生产生活条件。实施农村燃气发展规划，实行市场化运作，因地制宜推进农村燃气化。加快农产品市场体系建设。加快农村小城镇的规划建设，加快推进文明生态村和沼气建设，坚持走生产发展、生活富裕、生态良好的可持续发展之路。

深化农业产业结构调整，为农民增收创造条件。继续推进“五退五还”进程，全力推进4个10万亩生态林、花卉、优质林木、优质水果基地建设。大力发展农产品的精深加工和运销，加快水产品出口加工区建设进程。抓住海南无疫区建设的有利条件，以罗牛山为龙头，启动30万头种猪和100万头生猪饲养加工项目建设，加快农牧业的集约化、产业化。抓住我市罗非鱼养殖纳入全国优势水产品区域规划的契机，加快低产鱼塘改造，逐步扩大渔业出口生产规模。实施“24—1”工程，全市24个镇每年每个镇抓一个上规模的龙头特色经济项目，带动乡镇经济发展。提高农业科技含量，构建琼北瓜果菜种子基地，以种子种苗示范基地推进标准化、规模化农业的发展。建立农业应急防疫体系，严防死守，坚决打好预防禽流感阻击战，加快推进无规定疫病示范区建设，确保海口无疫区。完善农业社会化服务体系，全力抓好农业产前、产中、产后等服务工作。

稳定农村土地政策，继续推进扶贫工作。确保农民增收的根本在于让农民拥有足量的土地。因此，要依法稳定农村土地承包关系，保护农民的合法权益。实行严格的耕地保护制度，妥善解决农村土地纠纷问题。研究解决失地农民的生产、生活、就业问题。改进和创新扶贫工作，集中扶贫资金，帮扶重点贫困村镇，着力解决农村人畜饮水，发展农村沼气，建设村镇道路等基本建设问题。千名下乡工作队要在突出智力扶贫、教育扶贫、科技扶贫上下功夫。各单位要在项目、规划扶贫上下力气，帮助贫困乡镇引进项目，搞好发展规划。要广泛开展企业联手扶贫，抓好企业扶贫示范村试点，把试点引向由点到面，综合规划，突出重点，全面发展的扶贫之路，努力提高扶贫工作水平。

大力发展农村服务业，引导农村劳动力向二、三产业转移。农村劳动力向二、三产业转移，关键在于提高劳动技能，根本在于大力发展农村加工服务业。因此，要创办农民技术培训学校，鼓励各类职业技术学校和各种技能培训机构开展对农民的职业技能培训。要消除影响农村劳动力转移的体制性障碍，改革户籍管理制度，取消农民和外来工进城务工的各种限制。取消对农村个体工商户和私营企业的不合理限制，对农村个体工商户免收个体工商管理费。要保障进城就业农民的合法权益，建立农民工的综合服务机构，切实做好农民工的就业服务工作。要做好规划，充分利用中心城市的辐射作用，大力发展农产品加工运销业，吸纳更多的农村劳动力从事农产品的加工运销。大力发展农村多层次、多形式的服务业，积极组织农村劳动力向餐饮业、建筑业、商贸业、休闲业等转移。大力发展农村各种形式的中、小集市，安置农村剩余劳动力。

五、全面提高城市规划水平，加快基础设施建设，切实加强城市综合管理，进一步提升和改善海口城市形象

要抓住城市规划国际国内招标的有利时机，以规划为龙头，统筹城市建设和管理，加快热带滨海绿色国际性城市的建设步伐。

高水平完成城市规划，在规划上取得新突破。创新城市规划编制方法，广泛吸收专家和市民参与规划编制的全过程，高标准完成城市总体规划、“海口外滩”规划、西海岸规划、灯光环境规划的设计编制、修订工作。实施南渡江两岸、东海岸等区域性规划的国际招标。加快交通路网、地下管网、水系、生态环境等专业性规划编制。加快制定工业、农业、旅游、商贸、文化、教育等产业性专项规划。城市的主要规划要通过人大立法。要坚持规划的科学性、统一性、公开性、权威性，实施城市规划公示制，强化规划的法律权威。

高标准推进城市建设，在城建上取得新进展。全力推进“3366”工程建设、抓紧完成南海大道二期、白龙路、丘海大道等改造工程，启动加快迎宾大道、货运大道、滨江西路、海甸岛环岛路、海新大桥、海瑞大桥等项目建设，拉开城市骨架，完善城市道路网络，推进“五网”建设。启动城市水系规划建设，加快城市生态森林工程建设，全面启动全市绿化改造，继续推进“五大出口”生态森林建设，逐步形成城在绿中、城在花中，城在水中的生态城市格局。推进城市亮化工程，切实加快以城中村和老城区等为重点的旧城区改造步伐，基本完成停缓建工程处置。加快第二理想居住地建设步伐，切实抓好全国环保城市复检、最佳人居环境奖和国家绿色交通城市的创建工作，进一步提升城市品位，改善城市形象。

高效能实施城市管理，在城管上取得新成效。城市管理是政府工作的永恒主题。要坚持城市建设和管理并重，着力研究解决未来城市交通瓶颈、水源稀缺、能源短缺等城市通病，不断提高城市现代化的管理水平。要切实加强交通管理，整治交通“三乱”。要切实加强城市地下水资源管理，严格控制地下水源开采。采取坚决措施，抓好城市生活用水，地表水源的环境保护。认真研究制定城市未来能源供给的安全保障规划，为实现海口可持续发展提供资源、能源储备保障。要强化环境卫生管理，继续推进“八查十治”。要下大力气加强城市街道立面的规划管理，净化空间环境。要下决心整治城市广告，美化广告形象。要大力整治街道摊点、大排档，优化街道环境。要全面加强城市各项综合管理。

六、解放思想与时俱进，下大力气创新体制，加快建设公共服务型政府，不断增强经济社会发展的活力和动力

体制创新，是经济社会发展的不竭动力，是建设公共服务型政府的迫切要求。加快建设公共服务型政府，逐步建立与经济特区发展相适应，与产业特色、环境特色、开放特色相适应的更具活力、更加开放的行政管理体系。

创新行政管理体制，规范政府行政行为。全面贯彻执行《行政许可法》和市委《关于行政管理体制改革若干问题的决定》，不断完善行政管理体制。深化行政审批制度改革，简化审批程序，加紧组建行政审批中心，主要的行政审批要实行集中审批、联合审批，逐步实现行政统一审批。大力推进城市管理体制的改革创新，在完成环卫管理体制改革的基础上，在规划、城建、环保、园林、国土等部门，推行城市管理决策权、执行权、监督权三权分离的改革，实行简政放权，把更多的管理权限下放给区级政府。推进城市管理综合执法的试点改革，实施相对集中执法，逐步实现统一执法，尽快改变执法队伍政出多门、以罚代法、以罚代管的局面。按照全省的统一部署，认真抓好事业单位的改革。

不断深化国有企业改革，努力创新城市经营机制。积极探索国有资产监督和运营的有效形式，建立健全国有资产管理和监督体制。要把国有企业的产权推向国际国内两个市场，实现产权多元化。要突破国企改革的瓶颈，加快国有工业企业改革，完成椰树、椰岛的改革。加快农口国有企业的改革，完成罗牛山农场股份制规范化改造。高度重视和抓好琼山区国有企业的改革。加快城市国有公共事业管理企业的改革，自来水、污水处理、垃圾处理、园林绿化等公共事业要推向市场，逐步实现公共事业资产经营市场化运作，投资主体多元化、股份化。要突破城市经营体制瓶颈，加大城市公共资源的经营力度。坚定不移地推进出租车、机动车牌号、公交车线路、广告经营权的招标拍卖。实行城市公共事业基础设施市场化经营，探索城市地下管网的公开招标经营。推进城市基础设施投融资体制的改革，完善城市建设投资公司的经营体制，完成城市基础设施投资“政府入口、市场出口”的改革试验。

创新财政管理体制，深化农口各项改革。加快国库支付制度改革，逐步推行全市的国库支付。全面加强和完善全市会计中心管理体制，改革财政的结算方法和手段，转变财政结算职能。改革财政预算方法，逐步实现公共财政预算。改革财政监督方式，强化预算监督，深化审计监督，加强社会监督，严格执行财政预算。巩固扩大农村税费改革成果，逐步减免农村税赋，确保农民负担明显减轻不反弹，确保农村基层组织正常运转，特别是农村义务教育经费的正常需要。依法稳定农村土地承包关系，完善农村土地制度，按照依法、自愿、有偿的原则进行土地承包经营权流转，逐步实现集约化经营。深化水务管理体制改革，运用市场机制，逐步实现水务系统股份化运作，多元化经营。

七、不断扩大对外开放，加大招商引资力度，努力扩大社会需求，充分发挥“三大需求”在加快海口发展中的重大作用

在扩大开放中扩大投资、出口规模，不断增强经济发展动力。实施“走出去战略”，积极开展对内对外的经济技术交流。紧紧抓住海南航权开放试点机遇，开放外资准入领域，吸引国内外航空公司到海口设立办事处和基地，吸引国内外的客流和物流流向海口或在海口中转，以航权开放带动旅游、商贸、教育、医疗的对外开放。强化经济特区意识，全面实行产业开放。积极培育出口主体，筹建出口加工区。扶持中小企业开拓国际市场，努力扩大外贸出口。逐步提高出口对经济发展的拉动力。建立投资促进体系和管理服务体系，搞好服务。主动接轨华南经济圈，积极参与华南经济圈的经济技术合作和产业分工，使海口向更深层次、更广领域、更大范围开放。用扩大对外开放的理念，深化投资体制改革，扩大社会投资的规模和渠道，促进全社会投资的快速增长，力争全年完成全社会固定资产投资突破120亿元，比上年增长15%。加快美兰机场扩建工程建设；推进海口电厂技术改造工程建设；抓紧南渡江防洪工程和重点水库建设；加大市人民医院，市疾病防疫控制中心为重点的卫生基本建设；抓好最佳人居环境基础建设，适度扩大居住小区、商品房建设投资规模，提高建设的水平和质量，增强投资拉动力。

在经济发展中提高城乡居民收入水平，切实增强居民消费能力。要全面落实国家对城乡居民的各种税费减免政策，努力扩大就业，提高城乡居民的收入水平，探索党政机关以薪养廉分配方式的改革，逐步提高国家公务员的工资水平，增强城乡居民的消费能力。要把促进消费作为扩大内需的关键环节，着力改善消费结构。充分利用省会城市消费市场的优势，制定鼓励消费政策，引进投资，引进消费，扩大消费总量。围绕汽车、住房等消费热点，提高供给能力和服务质量。搞好节庆消费，加快消费结构升级。推动住房、汽车、通信、旅游、教育、休闲、体育、保健等消费继续走旺。

在扩大内外交流中搞活招商引资，着力增强发展活力。把招商的目光瞄准世界500强和国内500强企业。创新招商平台，充分利用博览会、交易会、推介会等多种平台，走出去招商。打好侨牌，引进侨资。(下转3B版)上接2A版)创新招商载体，实行园区整体招商、产业链招商，在国内外大力开展“药谷”、美安工业基地、“海口外滩”等项目招商、产业招商。创新招商手段，把走出去招商、规划招商、文化招商、以商招商、项目招商有机结合，吸引大项目、大企业落户海口，实现招商引资的新突破。抓住机遇，启动“今日无疫区，明日有商机”的大型农业招商活动，引进资金、技术、项目、人才，做大做强海口农业。

八、牢固树立科学发展观，坚持可持续发展战略，统筹社会事业协调发展，促进人与社会全面进步

加强教科文卫建设，协调发展各项社会事业。全面实施“科教兴市”战略，以科技创新为主线，以发展高新技术产业和科技兴农为重点，建立和完善以企业为主体、以高等院校和科研单位为依托的技术创新体系。切实加强信息化工作。实施人才工程纲要，建立产业发展与人才引进的柔性联动机制，用产业发展带动人才引进，以人才引进推进产业发展，营造尊重劳动、尊重知识、尊重人才、尊重创造的社会环境。大力发展教育事业，全面推进素质教育，扩大优质教育。统筹城乡、区域和各级各类教育协调发展，扶持一批农村中小学校建设，积极鼓励和支持办学基础较好的学校创建国家级、省级示范校，逐步形成名校示范、结构合理、特色鲜明、均衡发展的优质教育体系。实施文化精品战略，繁荣文艺创作，开展丰富多彩、健康向上的群众文体活动，力争全年重要节庆日都有高质量的文艺演出活动。加快推进文化体制改革，培育和发展有海口特色的文化产业。加强文化、体育设施建设，全面实施文化体育发展规划。加强农村卫生工作，完善社区卫生服务网络，健全城乡一体化的公共卫生体系和突发事件应急机制。高度重视人口与计划生育工作，加大计划生育投入，强化计划生育考核。继续搞好人民武装建设，创新“双拥”工作。认真做好民族宗教、外事侨务、对台事务、老年人、妇女儿童等工作。

大力推进就业再就业工程，健全社会保障体系。高度重视就业和再就业工作，创新工作思路，健全市、区、街道（镇）、社区的多层次就业再就业工作网络。加快发展服务业和劳动密集型产业，拓展就业渠道，扩大就业容量。各级政府要认真落实促进再就业各项政策，着力解决就业场地、贷款、税费等方面的问题。抓好就业培训和指导，帮助失业人员转变就业观念，提高职业技能。高度重视农民进城务工问题，着力解决“4050”人员和“四无”人员的就业。进一步完善社会保障体系，健全城乡医疗、养老、工伤保险体制，规范社会保险基金收支两条线管理。大力实施特困居民援助工程，健全市、区、街道（镇）、社区四级服务网络，着力解决特困群众就业、住房、就医、上学、养老、打官司等“六难”问题。

加强精神文明和民主法制建设，全力维护社会稳定。积极实施《公民道德建设实施纲要》，加强社会公德、职业道德、家庭美德教育。深入开展文明机关、文明社区、文明村镇、文明诚信企业等群众性创建活动，逐步形成良好的社会风气。坚持完善民主制度，坚持重大事项报告制度，自觉接受人大法律监督、政协民主监督、新闻舆论监督、群众社会监督，加强和规范对人大建议和政协提案的办理工作。大力推进依法行政，贯彻实施《行政许可法》，按期完成行政许可事项、规定和实施主体的清理工作，加快建立职责明确、行政规范、监督有效、保障有力的执法体制。完善社会信用服务体系，整顿和规范市场经济秩序。加强执法队伍建设。坚持群防群治的方针，深入开展“严打”斗争，加大打击“两抢”和黄赌毒力度，努力解决群众反映的热点难点问题，认真处理群众来信来访，全力维护社会稳定。高度重视安全生产，主动采取措施防止各类事故的发生。

九、牢固树立求真务实精神，加强政府自身建设，努力塑造务实创新勤政廉洁的政府形象

牢记宗旨，勤政为民。立党为公，执政为民，实现好、维护好、发展好人民群众的根本利益是政府工作的出发点和归宿。要牢固树立“群众利益无小事”的观念，把群众呼声和意愿作为指导工作的第一信号，把关心和服务群众作为第一职责，把群众的评价作为衡量工作政绩的第一尺度，着力解决涉及群众切身利益的突出问题。认真落实“三个善待”的理念，开展行政优质服务活动，切实解决企业、人民群众生产生活中的实际困难。继续落实为民办实事项目，今年确定的13项为民办实事，要明确责任，保证投入，确保年底兑现。

求真务实，开拓创新。要坚持科学的发展观和正确的政绩观，弘扬求真务实精神，大兴求真务实之风，强化执行意识，改进执行方式，加大执行力度，保证决策落实到位。改革政府绩效考核制度，把政府部门的工作作风、服务态度、服务质量和服务效率作为考核的重要内容。要深入基层，深入企业，深入群众，解决基层实际问题，继续完善现场办公制度，领导定点联系企业优质服务制度，以实实在在的工作业绩取信于人民，取信于企业，取信于社会。

廉洁从政，艰苦奋斗。要继续推进“四型”政府建设。各级政府和工作人员要牢记“两个务必”，始终保持谦虚、谨慎、不骄、不躁的作风，始终保持艰苦奋斗的作风。倡导讲实话，使实劲，办实事，求实效，反对形式主义、官僚主义和拜金主义。倡导埋头苦干，密切联系群众，反对奢侈浪费，脱离群众的作风。认真落实党风廉政建设责任制，加强廉政建设，坚持标本兼治，综合治理的方针，努力从源头上预防和治理腐败。加强公务员队伍建设，树立政府机关工作人员的良好形象。

各位代表，目标已确定，百舸已起航。历史赋予我们光荣而神圣的使命。为了完成今年的各项任务，我们必须振奋精神，埋头苦干；为了海口明天的现代化，我们必须克服任何困苦和艰难；为了海口人民明天的幸福美满，我们能够战胜任何风险和挑战。让我们团结在以胡锦涛同志为总书记的党中央周围，高举邓小平理论伟大旗帜，认真实践“三个代表”重要思想，在省委、省政府和市委的坚强领导下，团结和带领全市人民，解放思想，开拓创新，抢抓机遇，乘势而上，加快发展，为“做大做强做精做美”新海口，为全面实现“两个翻番，两个率先”的目标而努力奋斗！
